# The value of conventional radiographs for diagnosing internal fixation-associated infection

**DOI:** 10.1186/s12891-021-04170-3

**Published:** 2021-05-04

**Authors:** Cheng Li, Nora Renz, Andrej Trampuz, Cristina Ojeda-Thies

**Affiliations:** 1 Charité – Universitätsmedizin Berlin, corporate member of Freie Universität Berlin, Humboldt-Universität zu Berlin, and Berlin Institute of Health, Center for Musculoskeletal Surgery (CMSC), Mittelallee 3, 13353 Berlin, Germany; 2 Department of Traumatology and Orthopedic Surgery, Hospital Universitario 12 de Octubre, Avda Córdoba s/n, 28041 Madrid, Spain

**Keywords:** Prosthesis-related infections, Internal fixation, Radiography, Diagnostic imaging, Conventional radiography

## Abstract

**Background:**

The aim of the study is to  assess the diagnostic value of preoperative conventional radiographs for diagnosing infection associated with internal fixation devices.

**Methods:**

We prospectively collected data of patients undergoing removal of internal fixation devices for any reason. Infection was diagnosed in case of purulence, sinus tract, positive histopathology and/or positive peri-implant tissue or sonication fluid culture. In radiographs radiolucent lines, implant breakage or displacement, or periosteal reaction were assessed. White blood cell count (WBC) and serum C-reactive protein (CRP) were determined at admission.

**Results:**

We included 421 surgeries in 380 patients (median age 53.6 years, range 11–98 years), mainly indicated for infection (24.9%), nonunion (20.0%) and symptomatic implants (13.5%). Radiologic signs of infection included radiolucent lines (11.4%); implant breakage (12.4%) or displacement (10.7%); and periosteal reaction (7.1%). Infection was confirmed in 116 cases (27.6%). Only radiolucent lines (OR = 1.86 [95%CI: 1.00–3.38]) and periosteal reaction (OR = 2.48 [95%CI: 1.17–5.26]) were associated with infection, with a low sensitivity (16.4 and 12.1%, respectively), and high specificity (90.5 and 94.8%, respectively). Preoperative WBC and CRP had a sensitivity of 23.0 and 35.3%, and specificity of 91.7 and 89.5%, respectively.

**Conclusions:**

Radiological signs suggestive of infection were uncommon. Radiolucency and periosteal reaction were associated with infection, though with low sensitivity.

**Supplementary Information:**

The online version contains supplementary material available at 10.1186/s12891-021-04170-3.

## Background

Diagnosing internal fixation-associated infections preoperatively may be challenging, as investigation of body fluids such as synovial fluid in periprosthetic joint infection is not possible. Conventional radiographs are usually the first diagnostic investigation before surgery, including in suspected infection [1, 2]. However, their diagnostic accuracy for infection are limited compared to other imaging modalities such as computed tomography (CT) and magnetic resonance imaging (MRI), particularly in chronic low-grade infections [3]. Suggestive radiographic signs for infection after internal fixation are radiolucency, implant loosening, sequestration, and lack of progression of bone healing (i.e. nonunion), as well as periosteal bone formation, as determined on a consensus meeting of the international expert group on fracture-related infections defining confirmatory and suggestive criteria for infection [4, 5].

In this study we evaluated the diagnostic accuracy of individual findings of conventional radiographs for the diagnosis of infections after internal fixation, and  compared its performance with that of laboratory biomarkers such as preoperative serum C-reactive protein (CRP) concentration and white blood cell (WBC) count.

## Materials and methods

We reviewed consecutive patients in whom an orthopaedic internal fixation device (or part of it) initially inserted for any indication (fractures / elective orthopaedic surgery) was removed for any (presumed septic or aseptic) reason, between December 2014 and December 2017. Data was reviewed from a prospective database collected in a tertiary hospital. Internal fixation devices of any type were included, excluding arthroplasties and spinal and craniofacial fixations. Patients without recent conventional radiographs were excluded. Informed consent was obtained from all study participants included in this database study. In the case of minors (age below 18 years), the informed consent was obtained from a parent and/or legal guardian of the participant.

**Fig. 1:**
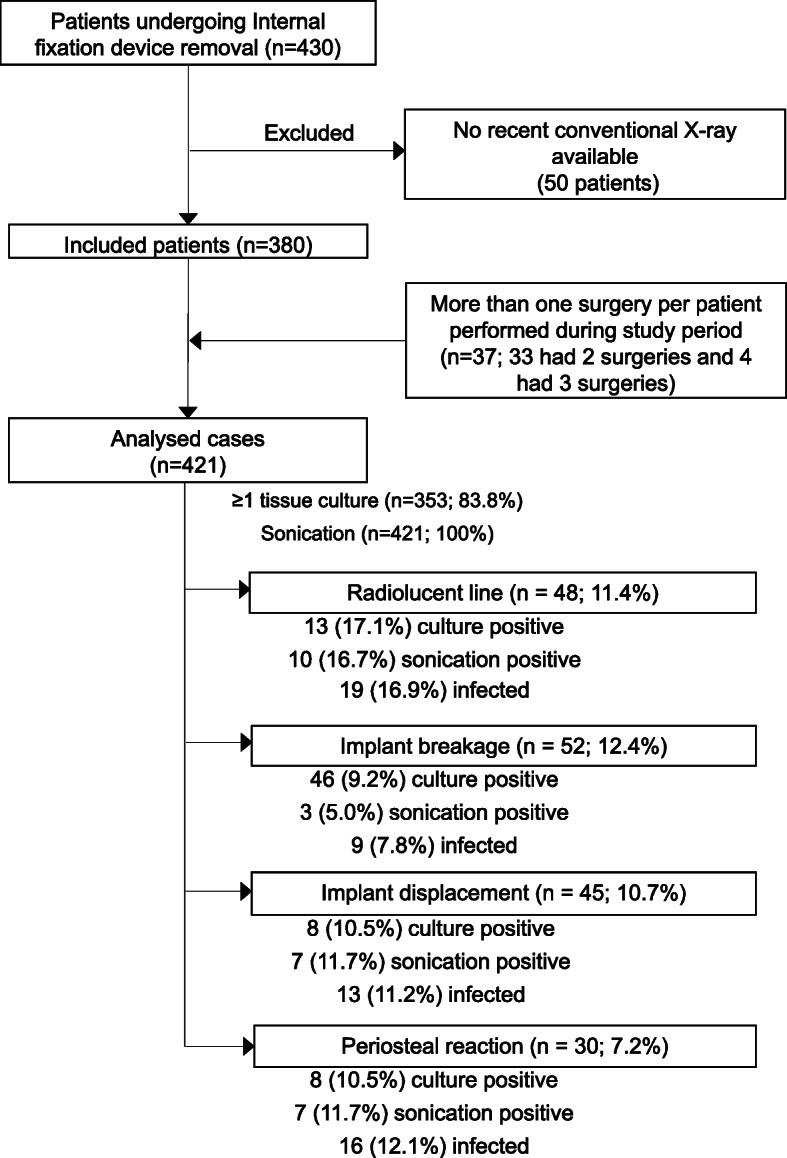
Patient selection and diagnostic flow chart

Internal fixation devices were removed in 473 surgeries from 430 patients. Recent radiographs were unavailable for 52 surgeries in 50 patients, mostly due to lack of digitization in the institution’s electronic health record among patients referred from other clinics. Thirty-three patients had two surgeries and four patients had three surgeries, resulting in 380 patients and 421 surgeries for analysis (Fig. 1). The term *case* indicates one surgery in one patient; thus, one patient could have several cases included for analysis.

We collected data regarding patient demography, index surgery, implant type, and revision surgery performed. Serum CRP concentration and WBC count within the week before surgery were documented, if available. Abnormal values were ≥ 10 mg/l for CRP levels and ≥ 11,000 leukocytes/mm^3^ for WBC count [6, 7].

**Fig. 2:**
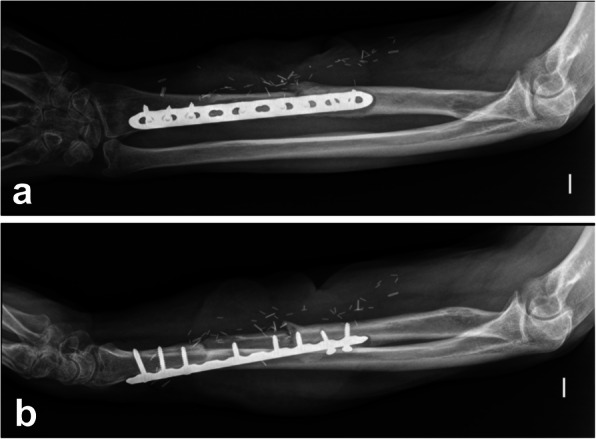
Postero-anterior (**a**) and lateral (**b**) radiographs of a chronic internal fixation related infection due to *Pseudomonas aeruginosa* following a Gustilo III-C open fracture of the radial diaphysis in a 62-year-old male. Note the radiolucent line surrounding the plate and the screw backout

**Fig. 3:**
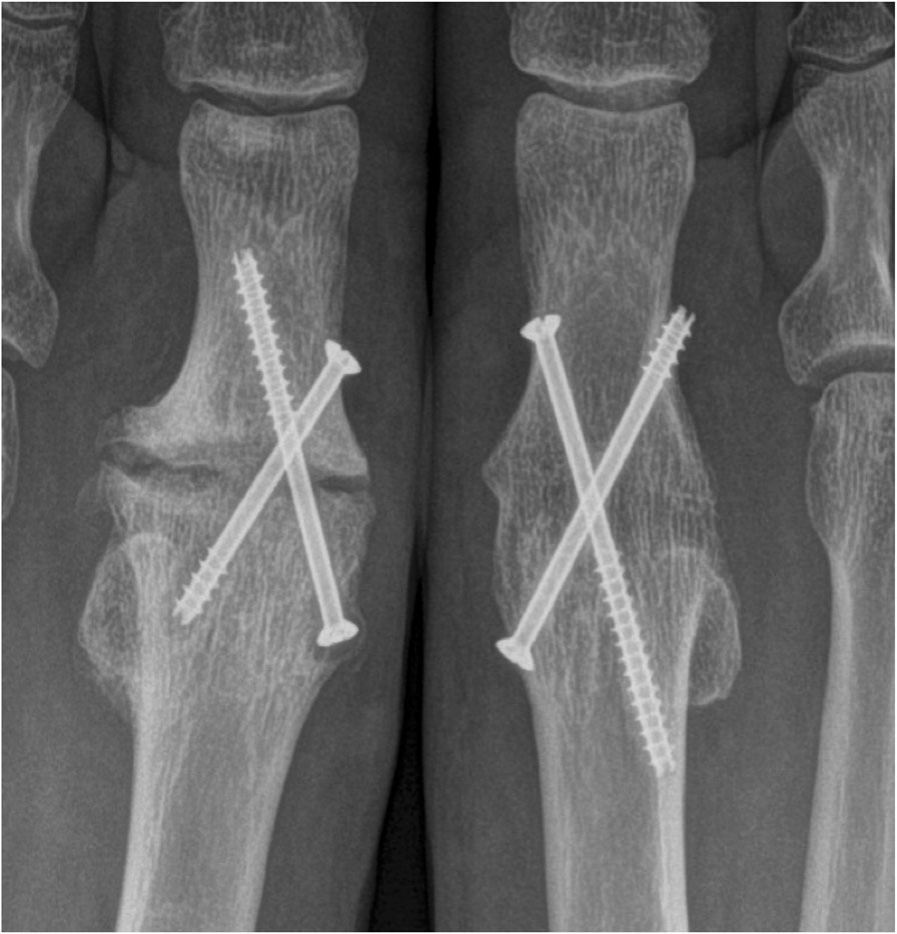
Detail of a postero-anterior standing radiograph of both feet following first metatarsophalangeal joint arthrodesis, in a 48 year-old female. Fusion of the hallux was achieved on the right foot but not on the left. Note the radiolucent line surrounding the implants in the metatarsal end of the fusion, and the periosteal reaction around the screw head. Revision surgery showed the presence of coagulase-negative staphylococci

Radiographs obtained up to 4 weeks before surgery were evaluated for osteolysis around the implant or at the fracture site (e.g. radiolucent lines of 2 mm of more or progression in successive radiographs) [8], implant displacement (e.g. screw back-out or migration), implant failure or breakage, and presence of periosteal reaction, as previously described [4, 5] (Figs. 2 and 3). Two orthopaedic surgeons, blinded to the patients’ infection status, analysed the radiographs independently. In case of disagreement, the images were re-examined until reaching an agreement.

An interdisciplinary team including an infectious diseases specialist and an orthopaedic surgeon classified each case. Implant-associated infection was defined with the presence of at least one of the following criteria: (i) purulence around the implant and/or a sinus tract communicating with the implant, and/or implant on view; (ii) histology showing inflammation in periimplant tissue [9, 10]; (III) positive culture of periimplant tissue or sonication fluid, as defined below. These criteria were adapted from the Infectious Diseases Society of America (IDSA) criteria for periprosthetic joint infections [11] used in the original trial protocol, in line with the recently proposed criteria for fracture-related infection [4]. Acute infections were defined as those less than four weeks postoperatively, or less than three weeks in acute haematogenous cases. Infections occurring beyond these periods were considered chronic. If infection was suspected preoperatively as per medical records, it was defined as "suggestive"; if infection could not be confirmed after surgery, it was analysed among the aseptic cases.

Periimplant tissue (bone or soft tissue) was obtained at the discretion of the operating surgeon. Tissue cultures were considered positive if (i) a highly virulent organism was identified by culture from ≥1 deep tissue sample or (ii) a phenotypically indistinguishable low-virulence pathogen [11, 12] was identified by culture from ≥2 deep tissue samples. Sonication of explanted devices was performed in the microbiology lab as part of standard procedure [13]. Sonication culture was considered positive if there was growth of ≥1 CFU/ml of a highly virulent organism or > 50 CFU/ml of a low-virulent organism [14].

### Statistical analysis

Sensitivity, specificity, positive predictive value (PPV), negative predictive value (NPV), accuracy, positive and negative likelihood ratio (PLR and NLR, respectively) and diagnostic odds ratio (DOR) of each diagnostic method (conventional radiology, laboratory markers, and microbiological tests) were calculated. For continuous variables, Receiver Operating Characteristic (ROC) curves were plotted and the Area Under the Curve (AUC) calculated, as well as Youden’s J Index, the threshold with a test’s maximum combined sensitivity and specificity. After confirming normal distribution, continuous variables were compared with Student’s t-test. Correlation of each diagnostic method with infection was analysed using chi-square tests. Subgroup analysis was performed for acute vs. chronic infections and for infected vs. aseptic nonunions. For variables with missing data such as tissue cultures, only cases with complete data were considered. Significance was set to *p* <  0.05 (two-sided). Data was analysed using IBM SPSS Statistics v. 20. The 2015 STARD guidelines [15] were used as reference for analysis and reporting of the results.

## Results

The patient median age was 53.6 (range, 11–98) years; 216 (56.8%) were male. Age and sex were similar for fracture vs. non-fracture cases, nonunion vs. consolidated cases, or acute vs. chronic infections. Table 1 summarises the baseline characteristics of the surgery performed. Infection was suspected preoperatively in 105 (24.9%) cases; 50 cases (11.9%) had a sinus tract. All retrieved implants were sonicated. An average of 2.5 ± 2.0 intraoperative deep tissue samples were obtained; ≥3 samples were obtained in 118 (44.7%) surgeries, and none were taken in 16.2%. Infection was confirmed in 116 cases (27.6%). In 26 cases (6.2%) preoperatively suspected infection could not be confirmed and were considered aseptic cases in the analysis.

**Table 1:** Baseline characteristics of the surgery performed

	All cases (*n* = 421)	Aseptic cases *(n* = 305)	Septic cases (*n* = 116)
Initial indication for surgery (n, %)			
- Fracture^a^	333 (79.1)	238 (78.0)	95 (81.9)
- Arthrodesis	37 (8.8)	28 (9.2)	9 (7.8)
- Osteotomy	33 (7.8)	26 (8.5)	7 (6.0)
- Tumor surgery	8 (1.9)	7 (2.3)	1 (0.9)
- Instability, ligament injury	5 (1.2)	3 (1.0)	2 (1.8)
- Other (limb lengthening, war injury)	5 (1.2)	3 (1.0)	2 (1.8)
Type of implant (n, %)			
- Plate and/or screws	325 (77.2)	239 (78.4)	86 (74.1)
- Intramedullary nail	71 (16.9)	52 (17.0)	19 (16.4)
- K-wires / Elastic nails / tension band	21 (5.0)	12 (3.9)	9 (7.8)
- Other (staple, anchor)	4 (1.0)	2 (0.6)	2 (1.8)
Indication for removal of internal fixation (n, %)			
- **Infection suspected**	**105 (24.9)**	**28 (9.2)**	**77 (66.3)**
○ Chronic infection^b^	72 (17.1)	22 (7.2)	50 (43.1)
○ Acute / haematogenous infection	33 (7.9)	6 (2.0)	27 (23.3)
**- Infection not suspected**	**316 (75.1)**	**277 (90.8)**	**39 (33.6)**
○ Nonunion^c^	84 (20.0)	74 (24.3)	10 (8.6)
○ Malposition, loss of fixation	57 (13.5)	48 (15.7)	9 (7.7)
○ Symptomatic hardware	57 (13.5)	52 (17.0)	5 (4.3)
○ Osteoarthritis	31 (7.4)	27 (8.9)	4 (3.4)
○ Avascular necrosis	24 (5.7)	20 (6.6)	4 (3.4)
○ Cutout	10 (2.4)	9 (3.0)	1 (0.9)
○ Periimplant fracture	19 (4.5)	18 (5.9)	1 (0.9)
○ Hardware breakage	14 (3.3)	14 (4.6)	0 (0.0)
○ Joint stiffness	9 (2.1)	7 (2.3)	2 (1.7)
○ Planned removal	7 (1.7)	6 (2.0)	1 (0.9)
○ Other (Charcot, tumor progression)	4 (1.0)	2 (0.7)	2 (1.8)
Anatomical location, AO region (n, %)			
- 1: Humerus, clavicle, scapula	81 (19.2)	54 (17.7)	27 (23.3)
- 2: Radius, ulna	41 (9.7)	31 (10.2)	10 (8.6)
- 3: Femur, patella	121 (28,7)	101 (33.1)	20 (17.2)
- 4: Tibia, fibula	130 (30.9)	88 (28.9)	42 (36.2)
- 6: Pelvis, acetabulum	9 (2.1)	5 (1.6)	4 (3.5)
- 7: Hand	1 (0.2)	0 (0.0)	1 (0.9)
- 8: Foot	38 (9.0)	26 (8.5)	12 (10.3)
Surgery performed (n, %)			
- Implant removal	185 (43.9)	109 (35.7)	76 (65.5)
- Reosteosynthesis	156 (37.1)	131 (43.0)	25 (21.6)
- Arthroplasty	51 (12.1)	46 (15.1)	5 (4.3)
- Masquelet, joint spacer	14 (3.3)	9 (3.0)	5 (4.3)
- External fixation for nonunion	6 (1.4)	2 (0.7)	4 (3.4)
- Dynamisation	4 (1.0)	4 (1.3)	0 (0.0)
- Arthrodesis	3 (0.7)	3 (1.0)	0 (0.0)
- Amputation	1 (0.2)	0 (0.0)	1 (0.9)
- Ligament reconstruction	1 (0.2)	1 (0.3)	0 (0.0)
Diagnosis of infection (n, %)			
- No infection	279 (66.3)	279 (91.5)	0 (0.0)
- Confirmed infection	116 (27.6)	0 (0.0)	116 (100.0)
- Suggestive infection	26 (6.2)	26 (8.5)	0 (0.0)
Radiographic changes (n, %)			
- Radiolucent line	48 (11.4)	29 (9.5)	19 (16.4)
- Implant breakage	52 (12.4)	43 (14.1)	9 (7.8)
- Implant displacement	45 (10.7)	32 (10.5)	13 (11.2)
- Periosteal reaction	30 (7.1)	16 (5.2)	14 (12.1)
- None	298 (70.8)	218 (71.5)	80 (69.0)

Note. Data are indicated as no. of cases (%)

^a^ Of the 333 fracture cases, 34 were open fractures (8.4% of the total series)

^b^ Chronic infection included non-unions in which infection was suspected

^c^ Non-unions without any suspicion of infection, ascertained by the medical history, clinical examination and tests

Table 2 summarizes the frequencies of the radiological and non-radiological findings, their sensitivity, specificity, PPV and NPV, likelihood ratios, and their DORs. Radiological findings had a low sensitivity, with acceptable specificity. Furthermore, it shows the performance of laboratory tests, tissue cultures and culture of sonication fluid as well as combined microbiology a. Radiographic findings were: periimplant radiolucency (48 cases, 11.4%), periosteal reaction (30 cases, 7.1%), and implant breakage or (52 cases, 12.4%) and displacement (45 cases, 10.7%). Only radiolucent lines (*p* = 0.047; OR = 1.9 [95% CI 1.0–3.5]) and periosteal reaction (*p* = 0.015; OR = 2.5 [1.2–5.3]) were associated with infection. Sensitivity of radiolucency and periosteal reaction were low (16,4% [95% CI: 10.2–24.4%] and 12,1% [95% CI: 6.1–18.4%], respectively), and specificity acceptable (90.5% [95% CI: 86.6–93.5%] and 94.8% [95% CI: 91.6–97.0%], respectively).

**Table 2:** Diagnostic performance of radiological and non-radiological findings for the diagnosis of infection (overall cohort)

Variable	Aseptic cases (n [total, %])^a^	Septic cases (n [total,%])^a^	Sensitivity (%, 95 CI)	Specificity (%, 95 CI)	PPV (%, 95 CI)	NPV (%, 95 CI)	Accuracy (%, 95 CI)	PLR	NLR	DOR (95% CI)	*p* value
**Radiological findings**											
Radiolucent line	29 (of 305 cases, 9.5)	19 (of 116 cases, 16.4)	16.4 (10.2–24.4)	90.5 (86.6–93.5)	39.6 (27.7–52.9)	74.0 (72.3–75.7)	70.1 (65.5–74.4)	1.72 (1.01–2.95)	0.92 (0.85–1.01)	1.86 (1.00–3.48)	0.047
Implant breakage	43 (305 cases, 14.1)	9 (116 cases, 7.8)	7.8 (3.6–14.2)	85.9 (81.5–89.6)	17.3 (9.5–29.4)	71.0 (69.6–72.4)	64.3 (59.6–69.0)	0.55 (0.28–1.09)	1.07 (1.00–1.15)	0.51 (0.24–1.09)	0.077
Implant displacement	32 (305 cases, 10.5)	13 (116 cases, 11.2)	11.2 (6.1–18.4)	89.5 (85.5–92.7)	28.9 (18.11 42.7)	72.6 (71.1–74.1)	67.9 (63.2–72.4)	1.07 (0.58–19.6)	0.99 (0.92–1.07)	1.08 (0.54–2.13)	0.832
Periosteal reaction	16 (305 cases, 5.2)	14 (116 cases, 12.1)	12.1 (6.8–19.4)	94.8 (91.6–97.0)	46.7 (30.6–63.4)	73.9 (72.5–75.3)	71.9 (67.4–76.2)	2.30 (1.16–4.56)	0.93 (0.86–1.00)	2.48 (1.17–5.26)	0.015
**Non-radiological findings**											
Increased WBC count^b^	16 (193 cases, 8.3)	21 (93 cases, 22.6)	22.6 (14.6–32.4)	91.7 (86.3–95.5)	56.8 (41.8–70.6)	71.1 (68.6–73.4)	69.2 (63.5–74.5)	2.72 (1.49–4.97)	0.84 (0.75–0.95)	3.2 (1.6–6.5)	0.001
Increased CRP value^c^	32 (193 cases, 16.6)	41 (93 cases, 44.1)	44.1 (33.8–54.8)	83.4 (77.4–88.4)	56.2 (46.4–65.4)	75.6 (71.9–78.9)	70.6 (65.0–75.8)	2.66 (1.80–3.93)	0.67 (0.55–0.81)	3.97 (2.27–7.69)	< 0.0001
Positive tissue cultures	37 (239 cases, 15.5)	94 (111 cases, 84.7)	84.7 (76.6–90.8)	84.5 (79.3–88.7)	71.8 (65.2–77.5)	92.2 (88.4–94.7)	84.6 (80.4–88.2)	5.47 (4.03–7.43)	0.18 (0.12–0.28)	30.19 (16.17–56.36)	< 0.0001
Positive sonication culture	89 (305 cases, 29.2)	107 (116 cases,92.2)	92.2 (85.8–96.4)	70.8 (65.4–75.9)	54.6 (50.0–59.1)	96.0 (92.7–97.8)	76.7 (72.4–80.7)	3.16 (2.63–3.79)	0.11 (0.06–0.21)	28.9 (14.0–59.5)	< 0.0001
Combined microbiology^d^	0	112 (116 cases, 96.6)	96.6 (91.4–99.1)	99.3 (97.7–99.9)	100.0	98.7 (96.7–99.5)	99.1 (97.6–99.7)	–	0.03 (0.01–0.09)	15,275.0 (815.8–286,001.6)	< 0.0001

NOTE. *PPV* Positive predictive value, *NPV* Negative predictive value, *PLR* Positive likelihood ratio, *NLR* Negative likelihood ratio, *DOR* Diagnostic odds ratio

^(a)^ The denominator of total cases for each comparison is given in parentheses

^(b)^ White blood cell (WBC) count ≥11.000/ mm^3^

^(c)^ C-reactive protein (CRP) value ≥10 mg/l

^(d)^ Combination of sonication and tissue cultures

Preoperative blood analysis was performed in the week preceding surgery in 286 (67.9%) cases. WBC counts were higher among infected patients (8903 ± 3.144 vs. 7399 ± 2574 leukocytes/mm3; *p* < 0.001), as well as CRP values (38.4 ± 66.8 mg/l vs. 10.2 ± 34.1; p < 0.001). The presence of infection was associated with increased WBC (*p* = 0.001; DOR = 3.2 [1.6–6.5]), but elevated CRP concentrations were more useful (*p* < 0.0001; DOR = 4.0 [2.3–7.7]). The AUC for the WBC count and the CRP value were 0.639 and 0.697, respectively. Youden Indexes were highest at ≥7.120 leukocytes/mm3 for WBC count (Youden = 0.23; 68.9% sensitivity and 54.1% specificity), and ≥ 4.25 ml/l for CRP concentrations (Youden = 0.32; 68.8% sensitivity and 63.2% specificity).

Tissue cultures were positive in 94 infected cases (sensitivity 84.7%, specificity 84.5%), while a positive sonication culture increased the odds of infection (DOR = 28.9; sensitivity 92.2%, specificity 70.8%). The combination of tissue cultures and sonication yielded the highest sensitivity (96.6%) and diagnostic accuracy (99.1%) of any of the tests evaluated. The number of tissue samples taken was higher among cases in which infection was confirmed than in aseptic cases (3.3 [SD 2.1] samples vs. 2.2 [SD 1.8] samples). Less than three samples were taken in 233 cases (55.3%); infection was confirmed in 43 of these, in 23 based on sonication fluid cultures. Of the 68 cases without tissue samples, five were considered infected (one based on the presence of a sinus tract, and four based on sonication fluid culture). Histological data was not available for analysis; however, no cases were diagnosed based on histological reports alone.

### Acute vs. chronic infections

Of the diagnosed infections, 30 (25.9%) were considered acute, and 86 (74.1%) chronic. Radiologic findings were more common in chronic infections (Table 2), but only radiolucency and periosteal reactions were significant, with a slightly higher sensitivity (20.9 and 15.1%, respectively; Table, Supplemental Digital Content 1). We could not find associations between radiologic findings and acute infection, though the number of cases with observed findings was very low. Diagnostic accuracy of non-radiological findings (laboratory and microbiology tests) was lower for chronic infections than for acute infections (Tables, Supplemental Digital Content 1 and 2). Average CRP values were higher in acute infections (84.4 ± 98.7 [acute infections] vs. 23.8 ± 42.1 mg/l [chronic infections]; *p* < 0.001), as were WBC counts (11.110 ± 3.361 vs. 7.969 ± 2.551 leukocytes/mm3; p < 0.001). Though CRP values were higher in chronic infections than aseptic cases (*p* = 0.008), WBC counts were not significantly different (*p* = 0.167). The AUC was higher in acute infections (0.808 for WBC and 0.873 for CRP); cutoffs with maximum Youden Indexes for acute infections were ≥ 9.390 leukocytes/mm3 for the WBC count (Youden = 0.50; 72.7% sensitivity and 77.7% specificity), and ≥ 8.95 mg/l for CRP concentration (Youden = 0.64; 83.3% sensitivity and 80.3% specificity).

### Nonunions

Of 98 nonunions included, 17 were considered infected. Nonunion was not more common in infected (14.7%) than in noninfected cases (26.6%). All radiologic findings were more common in nonunion cases than in consolidated cases (radiolucency 22.4% vs. 8.0%, implant breakage 27.6% vs. 7.7%, implant displacement 14.3% vs. 9.6%, periosteal reaction 15.3% vs. 4.6%, respectively). Only radiolucency and periosteal reaction remained significant  when comparing septic vs. aseptic nonunions (Table, Supplemental Digital Content 3), with a higher sensitivity than in other analyses (41.2 and 35.3%, respectively). Elevated WBC counts were not associated with infected nonunion. Though CRP levels were higher in infected nonunions, sensitivity was low (46.1%).

### Fractures vs. non-fracture cases

No differences were found regarding radiologic findings when comparing cases in which the initial indication for internal fixation was a fracture, compared to non-fracture cases (periimplant radiolucency 10.2% vs. 15.9% [*p* = 0.067], implant breakage 11.7% vs. 14.8% [*p* = 0.219], implant displacement 11.4% vs. 8.0% [*p* = 0.176], periosteal reaction 6.3% vs. 8.0% [*p* = 0.290], respectively). A subanalysis of the 333 cases in which the initial indication was fracture lost significance for radiologic findings on any type, though analysis was underpowered after removing over one fifth of cases. Similar results and limitations were observed when analysing only certain implant types, such as plates and/or screws.

## Discussion

Though consensus guidelines include radiographic findings after internal fixation, the diagnostic value of these findings for infection has not been studied. We found that radiolucency surrounding the implant or periosteal reactions were associated with internal fixation-related infection, while implant breakage or displacement showed no association. Though radiological signs were quite specific, sensitivity was low, suggesting that normal radiographs cannot rule out infection. Radiolucency and periosteal reaction were more likely to be observed in infected cases (PLR 1.7 and 2.3, respectively), particularly in chronic infections (PLR 2.2 and 2.9, respectively) and infected nonunions (PLR 2.2 and 3.2, respectively).

While the consensus group proposed the term “fracture-related infection” [4], the term “internal fixation-related infection” may be more appropriate, as about one fifth of our cases were fixations performed as elective procedures (such as the case described in Fig. 3). Other authors used the term “Device-related infections” in contrast to prosthetic joint infections, grouping fracture- and non-fracture cases [16]. No differences were found when evaluating fracture vs. non-fracture cases or cases with a certain type of implant, such as plates and/or screws, or intramedullary nails.

Patients with acute infections or with revision surgery shortly after initial surgery (due to fracture malposition or as planned removal) may not have developed radiological changes yet. Significance was lost when examining radiographs in acute cases. On the other hand, laboratory and microbiological tests had a higher diagnostic accuracy in acute infections; these are usually not as much a diagnostic challenge as chronic infections since they are most commonly caused by virulent pathogens. Most diagnosed infections were chronic infections suspected preoperatively, or found after revision surgery for indications such as nonunion, osteoarthritis, or removal of symptomatic devices. In these, the time between implant insertion and removal was long enough for these infections to be considered chronic.

Radiological findings significantly associated with infection (periimplant radiolucency, periosteal reaction) reflect changes in the bone itself, while implant-related findings (breakage, displacement) were non-significant. Twenty-six cases in which infection was clinically suspected (nonunion, inflammation, etc.) were considered aseptic according to the predefined criteria used for this study. Clinical signs suggestive of infection are nonspecific, and the use of validated criteria including microbiology has improved diagnostic accuracy of implant-associated infections [5]. Finally, intraoperative tissue samples were obtained at the surgeons’ discretion, conditioned by the preoperative indication for implant removal. Less than three tissue samples were taken in 55.3% of cases, and none in 16.2%.

We cannot exclude having missed some cases of implant-associated infection. We were unable to follow the 279 cases in which infection was excluded, and some cases could have developed infection following the revision surgery: this could be because the infection was always present (e.g. a false negative), or because it appeared newly as a complication following the revision surgery. However, the criteria used are based on diagnostic criteria sufficiently validated elsewhere [4, 11], and the lack of positive cultures in patients in whom infection was not suspected preoperatively strongly suggest absence of infection. Of the cases in which less than the three tissue samples were taken, over half of the infections were diagnosed based on sonication alone; we believe standardized sonication of all retrieved implants, regardless of surgical indication, can detect infections that would otherwise go undetected. The strength of this study lies in that it involves one of the largest cohorts of internal-fixation-related infections recorded to date. The surgeons evaluating the radiographs were blinded to the infection status, and the definition of infection was in accordance with internationally accepted guidelines [4, 5, 11].

Previous studies analysed radiological signs in orthopaedic infections including prosthetic joint infections, spinal infections, and paediatric acute haematogenous osteomyelitis; cases were included based on the suspicion of infection. We are not aware of any study analysing all internal fixation device retrievals, regardless of indication, as would be necessary to study diagnostic accuracy. In 1985, Al-Sheikh [17] compared bone scintigraphy and radiography for diagnosing bone infections in 21 orthopaedic patients, reporting 60% sensitivity and 67% specificity. Tumeh [18] examined radiographs in 104 patients with chronic osteomyelitis following fractures, surgery or infection, in search of bone destruction (erosion, lucency around implants), periosteal reaction, sequestra, soft-tissue swelling, cartilage-space narrowing, and changes from prior examinations, with a 37% sensitivity and 84% specificity.  They found some parameters were difficult to recognize and could reflect reactions to the surgery or the fracture itself. In 11 patients evaluated for the presence of spine infection using radiographs and bone scintigraphy, Whalen [19] reported 78% sensitivity and 100% specificity. In a more recent series of paediatric patients with acute haematogenous osteomyelitis, the sensitivity and specificity of early X-rays (performed at admission) were 16 and 96% [20] (Table 3). The relative sensitivity and specificity of some of these studies can also be affected by small sample sizes, leading to wide confidence intervals, and a certain level of selection bias due to their inclusion criteria.

**Table 3:** Sensitivity and specificity of conventional radiographs reported by other authors and in the current series

	Infections analyzed	Cases with / without disease	Sensitivity (95% CI)	Specificity (95% CI)
Al-Sheikh (1985) [17]	Chronic orthopedic, with and without implants	10 / 12	60% (28–86%)	76% (36–89%)
Tumeh, (1987) [18]	Chronic osteomyelitis after fracture, infection or surgery (excl. Joint prosthesis)	35 / 69	37% (21–55%)	84% (73–92%)
Whalen (1991) [19]	Spinal	9 / 2	78% (40–96%)	100% (16–100%)
Malcius (2009) [20]	Acute hematogenic, pediatric	156 / 27	16% (10–23%) (early)	96% (78–100%) (early)
Current series	Internal fixation devices	116 / 305	RL^a^: 16% (10–24%)	RL^a^: 91% (87–94%)
			PR^a^: 12% (7–19%)	PR^a^: 95% (92–97%)

^a^*RL* Radiolucent line, *PR* Periosteal reaction

Periprosthetic bone loss has been hypothesized to be mediated through osteoclast activation over several pathways originating in cytokine expression by neutrophil granulocytes [21]. Local bone loss leads to loss of mechanical stability and of fixation, observed as displacement of internal fixation and screw backout, which can lead to implant failure and breakage. The periosteal reaction observed in bone infections is due to the elevation of the periosteum with increased pressure. In our series, nonunions were not more common in septic vs. aseptic cases; though infection may impede fracture healing, the sole presence of a nonunion could not be used as a marker for infection. Radiolucency and periosteal reaction remained associated with infection in nonunions, with a higher sensitivity than for the overall analysis.

We do not analyse erythrocyte sedimentation rates (ESR) routinely anymore in our insitution, as it was replaced by newer markers such as CRP levels, reported to be more useful for the diagnosis of osteoarticular infection [22]. Other studies described a similar accuracy of serum markers; inclusion of different types of musculoskeletal infection (osteomyelitis, periprosthetic infection, acute/chronic infection, etc.) and cut-off points [22, 23] limit comparability. The pattern observed in our study was similar to another study analysing laboratory markers in nonunions [24]. Another study also observed a limited value of serum inflammatory markers for diagnosing fracture-related infections [25], with a sensitivity of 38% (using a threshold of only 5.0 mg/l); the AUC was nearly identical to our study.

The diagnostic accuracy of sonication and tissue cultures for fracture-related infection in contrast to prosthetic joint infection has been an area of recent interest [2, 16, 26]. A systematic review by Onsea et al. [26] showed that evidence was scarce, and lower for sonication alone than for tissue cultures, in contrast to our study, though other authors cultured a minimum of three tissue samples [16]. As shown in previous studies, the combination of sonication and tissue cultures yielded the highest diagnostic accuracy [16, 27–30]. Though antibiotics should be withheld prior to microbiological sampling [4], culture of sonicate fluid could improve the diagnostic accuracy under antimicrobial pressure [27]. Biofilm-associated infections elicit a reaction from bone, even under suppressive antimicrobial treatment [31].

Our study has several limitations. First, the histopathological definition criteria (i.e. presence of ≥5 granulocytes per high-powered field [32]) was implemented later during the study, therefore, histopathology findings are missing in most cases. The index test (radiography) was performed within four weeks of the reference test (confirmation of infection). Second, 50 patients (9.7% of the initial cohort) were excluded due to lack of conventional X-rays within four weeks before surgery. Furthermore, serial radiographs were not analysed in our series, as they were unavailable in many cases that consulted for implant removal and were not managed initially in our centre. Third, only about two thirds of patients had laboratory tests from the week before surgery, as we receive referrals from a wide geographic area and data is not always included in our electronic health records. Fourth, we chose not to include findings such as soft tissue swelling or sequestration because of the subtleness of the former and the superposition of the metallic implants in the latter. Fifth, other imaging modalities such as CT and MRI were not routinely performed prior to implant removal, due to radiation dose and costs. Therefore, we are unable to assess their diagnostic accuracy in this setting. We believe ordering updated conventional X-rays and laboratory tests to all patients presenting for implant removal could increase the likelihood of detecting implant-associated infection; comparison of serial radiographs and using more advanced imaging modalities would increase diagnostic accuracy and improve upon the values observed in our study, taking into account the limitations mentioned.

In conclusion, radiologic signs of infection are uncommon, even in cases of confirmed infection. Recent conventional X-rays should be reviewed prior to implant removal in search of subtle radiographic changes. In particular, (a) radiolucency surrounding the implant and (b) the presence of a periosteal reaction were associated with infection, with a low sensitivity and high specificity, making them useful for ruling in patients in whom infection is likely. However, though they are less common in non-infected cases, the absence of these radiological changes does not rule out infection. Therefore, we recommend obtaining (c) tissue samples for culture and histology and (d) sonication of the retrieved implants if infection is suspected, especially if new implants are to be used in the revision surgery.

## Supplementary Information

**Additional file 1: Table, Supplementary Digital Content 1**: Diagnostic accuracy of radiological and non-radiological findings for the diagnosis of chronic infection.

**Additional file 2: Table, Supplementary Digital Content 2**: Diagnostic accuracy of radiological and non-radiological findings for the diagnosis of acute infection.

**Additional file 3: Table, Supplementary Digital Content 3**: Diagnostic accuracy of radiological and non-radiological findings for the diagnosis of infected nonunion.

## Data Availability

Upon request to the corresponding author, following institutional policy.
