# Supplementary material for: The value of conventional radiographs for diagnosing internal fixation-associated infection
Source: BMC Musculoskelet Disord. 2021 May 4;22:411. doi: 10.1186/s12891-021-04170-3 (PMC8097790; doi:10.1186/s12891-021-04170-3)
Supplement: Supplementary file 1 — Additional file 1: Table, Supplementary Digital Content 1: Diagnostic accuracy of radiological and non-radiological findings for the diagnosis of chronic infection. [file 12891_2021_4170_MOESM1_ESM.docx]

**TABLE, SUPPLEMENTAL DIGITAL CONTENT 1:** Diagnostic accuracy of radiological and non-radiological findings for the diagnosis of chronic infection.

| Variable | Aseptic cases  (n=305)^1^ | Chronic infections  (n=86)^1^ | Sensitivity  (%, 95% CI) | Specificity  (%, 95% CI) | PPV  (%, 95% CI) | NPV  (%, 95% CI) | Accuracy  (%, 95% CI) | PLR | NLR | DOR (95% CI) | P value |
| --- | --- | --- | --- | --- | --- | --- | --- | --- | --- | --- | --- |
| **Radiological findings** | | | | | | | | | | | |
| Radiolucent line | 29 | 18 | 20.9 (12.9 – 31.1) | 90.5 (86.6 – 93.5) | 38.3 (26.6 – 51.5) | 80.2 (78.4 – 82.0) | 75.2 (70.6 – 79.4) | 2.20 (1.29 – 3.77) | 0.87 (0.78 – 0.98) | 2.52 (1.32 – 4.80) | 0.004 |
| Implant breakage | 43 | 8 | 9.3 (4.1 – 17.5) | 85.9 (81.5 – 89.6) | 15.7 (8.3 – 27.6) | 77.1 (75.6 – 78.5) | 69.1 (64.2 – 73.6) | 0.66 (0.32 – 1.35) | 1.06 (0.97 – 1.15) | 0.62 (0.28 – 1.39) | 0.243 |
| Implant displacement | 32 | 11 | 12.8 (6.6 – 21.7) | 89.5 (85.5 – 92.7) | 25.6 (15.3 – 39.5) | 78.5 (76.9 – 79.2) | 72.6 (67.9 – 77.0) | 1.22 (0.64 – 2.32) | 0.97 (0.89 – 1.07) | 1.25 (0.60 – 2.60) | 0.547 |
| Periosteal reaction | 16 | 13 | 15.1 (8.3 – 24.5) | 94.6 (91.6 – 97.0) | 44.8 (28.9 – 61.9) | 79.8 (78.3 – 81.3) | 77.2 (72.8 – 81.3) | 2.88 (1.44 – 5.75) | 0.90 (0.82 – 0.98) | 3.22 (1.48 – 6.99) | 0.002 |
| **Non-radiological findings** | | | | | | | | | | | |
| Increased WBC count^2^ | 13 (193 cases) | 7 (69 cases) | 10.1 (4.2 – 19.8) | 93.3 (88.8 – 96.4) | 35.0 (18.3 – 56.4) | 74.4 (72.7 – 76.0) | 71.4 (65.5 – 76.8) | 1.51 (0.63 – 3.62) | 0.96 (0.88 – 1.05) | 1.6 (0.6 – 4.1) | 0.427 |
| Increased CRP level^3^ | 32 (193 cases | 25 (69 cases) | 36.2 (25.0 – 48.7) | 83.4 (77.4 – 88.4) | 43.9 (33.4 – 54.9) | 78.5 (75.2 – 81.6) | 71.0 (65.1 – 76.4) | 2.19 (1.40 – 3.41) | 0.76 (0.63 – 0.92) | 2.86 (1.54 – 5.32) | 0.001 |
| Positive tissue cultures | 37 (239 cases) | 64 (81 cases) | 79.0 (68.5 – 87.2) | 84.5 (79.3 – 88.6) | 63.4 (55.8 – 70.4) | 92.2 (88.6 – 94.8) | 83.1 (78.6 – 87.1) | 5.10 (3.72 – 7.01) | 0.25 (0.16 – 0.38) | 20.6 (10.8 – 39.0) | < 0.0001 |
| Positive sonication culture | 89 | 77 | 89.5 (81.6 – 95.1) | 70.8 (65.4 – 75.9) | 46.4 (41.7 – 51.1) | 96.0 (92.8 – 97.8) | 74.9 (70.3 – 79.2) | 3.07 (2.54 – 3.71) | 0.15 (0.08 – 0.28) | 20.7 (10.0 – 43.2) | < 0.0001 |
| Combined microbiology^4^ | 0 | 82 | 95.4 (88.5 – 98.7) | 100.0 (98.8 – 100.0) | 100.0 | 98.7 (96.7 – 99.5) | 98.9 (97.4 – 99.7) | - | 0.05 (0.02 – 0.12) | 11,201.7 (597.0 – 210,179.3) | < 0.0001 |

NOTE. PPV = Positive predictive value; NPV = Negative predictive value; PLR = Positive likelihood ratio; NLR = Negative likelihood ratio; DOR = Diagnostic odds ratio.

^(1)^ Where not otherwise indicated

^(2)^ White blood cell (WBC) count ≥ 11.000/ mm^3^

^(3)^ C-reactive protein (CRP) level ≥ 10 mg/l

^(4)^ Combination of sonication and tissue cultures
