# Supplementary material for: The value of conventional radiographs for diagnosing internal fixation-associated infection
Source: BMC Musculoskelet Disord. 2021 May 4;22:411. doi: 10.1186/s12891-021-04170-3 (PMC8097790; doi:10.1186/s12891-021-04170-3)
Supplement: Supplementary file 2 — Additional file 2: Table, Supplementary Digital Content 2: Diagnostic accuracy of radiological and non-radiological findings for the diagnosis of acute infection. [file 12891_2021_4170_MOESM2_ESM.docx]

**TABLE, SUPPLEMENTAL DIGITAL CONTENT 2:** Diagnostic accuracy of radiological and non-radiological findings for the diagnosis of acute infection.

| Variable | Aseptic cases  (n=305)^1^ | Acute infections  (n=30)^1^ | Sensitivity  (%, 95% CI) | Specificity  (%, 95% CI) | PPV  (%, 95% CI) | NPV  (%, 95% CI) | Accuracy  (%, 95% CI) | PLR | NLR | DOR (95% CI) | P value |
| --- | --- | --- | --- | --- | --- | --- | --- | --- | --- | --- | --- |
| **Radiological findings** | | | | | | | | | | | |
| Radiolucent line | 29 | 1 | 3.3 (0.1 – 17.2) | 90.5 (86.6 – 93.5) | 3.3 (0.5 – 19.6) | 90.5 (89.8 – 91.1) | 82.7 (78.2 – 86.6) | 2.20 (1.29 – 3.77) | 0.35 (0.05 – 2.48) | 0.32 (0.04 – 2.50) | 0.223 |
| Implant breakage | 43 | 1 | 3.3 (0.1 – 17.2) | 85.9 (81.5 – 89.6) | 2.3 (0.3 – 14.0) | 90.0 (89.3 – 90.7) | 78.5 (73.7 – 82.8) | 0.24 (0.03 – 1.66) | 1.13 (0.04 – 1.22) | 0.21 (0.03 – 1.58) | 0.072 |
| Implant displacement | 32 | 2 | 6.7 (0.8 – 22.1) | 89.5 (85.5 – 92.7) | 5.9 (1.6 – 19.9) | 90.7 (89.8 – 91.5) | 82.1 (77.6 – 86.1) | 0.64 (0.16 – 2.52) | 1.04 (0.94 – 1.16) | 0.61 (0.14 – 2.68) | 0.391 |
| Periosteal reaction | 16 | 1 | 3.3 (0.1 – 17.2) | 94.8 (91.6 – 97.0) | 5.9 (0.9 – 31.3) | 90.9 (90.3 – 91.5) | 86.6 (82.4 – 90.0) | 0.64 (0.09 – 4.63) | 1.02 (0.95 – 1.10) | 0.62 (0.08 – 4.87) | 0.538 |
| **Non-radiological findings** | | | | | | | | | | | |
| Increased WBC count^2^ | 13 (193 cases) | 10 (24 cases) | 41.7 (22.1 – 63,4) | 93.3 (88.8 – 96.4) | 43.5 (27.5 – 60.9) | 92.8 (90.2 – 94.8) | 87.6 (82.4 – 91.6) | 6.19 (3.05 – 12.54) | 0.63 (0.45 – 0.88) | 9.9 (3.7 – 26.6) | < 0.0001 |
| Increased CRP level^3^ | 32 (193 cases | 16 (24 cases) | 66.7 (44.7 – 84.4) | 83.4 (77.4 – 88.4) | 33.3 (24.7 – 43.3) | 95.3 (91.9 – 97.3) | 81.6 (75.7 – 86.5) | 4.02 (2.63 – 6.15) | 0.40 (0.23 – 0.71) | 10.06 (3.97 – 25.49) | < 0.0001 |
| Positive tissue cultures | 37 (239 cases) | 29 (30 cases) | 96.7 (82.8 – 99.9) | 84.5 (79.3 – 88.6) | 43.9 (36.7 – 51.5) | 99.5 (96.7 – 99.9) | 85.9 (81.1 – 89.8) | 6.24 (4.61 – 8.46) | 0.04 (0.01 – 0.27) | 216.96 (28.39 – 1,657.87) | < 0.0001 |
| Positive sonication culture | 89 | 29 | 96.7 (82.8 – 99.9) | 70.8 (65.4 – 75.9) | 24.6 (21.3 – 28.2) | 99.5 (96.9 – 99.9) | 73.1 (68.1 – 77.8) | 3.31 (2.75 – 3.99) | 0.05 (0.01 – 0.32) | 70.38 (9.44 – 524.62) | < 0.0001 |
| Combined microbiology^4^ | 0 | 29 | 96.7 (82.8 – 99.9) | 100.0 (98.8 – 100.0) | 100.0 | 99.7 (97.8 – 100.0) | 99.7 (98.3 – 100.0) | - | 0.03 (0.00 – 0.23) | 12,016.3 (478.7 – 301,618.8) | < 0.0001 |

NOTE. PPV = Positive predictive value; NPV = Negative predictive value; PLR = Positive likelihood ratio; NLR = Negative likelihood ratio; DOR = Diagnostic odds ratio.

^(1)^ Where not otherwise indicated

^(2)^ White blood cell (WBC) count ≥ 11.000/ mm^3^

^(3)^ C-reactive protein (CRP) level ≥ 10 mg/l

^(4)^ Combination of sonication and tissue cultures
