# Supplementary material for: The value of conventional radiographs for diagnosing internal fixation-associated infection
Source: BMC Musculoskelet Disord. 2021 May 4;22:411. doi: 10.1186/s12891-021-04170-3 (PMC8097790; doi:10.1186/s12891-021-04170-3)
Supplement: Supplementary file 3 — Additional file 3: Table, Supplementary Digital Content 3: Diagnostic accuracy of radiological and non-radiological findings for the diagnosis of infected nonunion. [file 12891_2021_4170_MOESM3_ESM.docx]

**TABLE, SUPPLEMENTAL DIGITAL CONTENT 3:** Diagnostic accuracy of radiological and non-radiological findings for the diagnosis of infected nonunion.

| Variable | Aseptic nonunion  (n=81)^1^ | Infected nounion  (n=17)^1^ | Sensitivity  (%, 95% CI) | Specificity  (%, 95% CI) | PPV  (%, 95% CI) | NPV  (%, 95% CI) | Accuracy  (%, 95% CI) | PLR | NLR | DOR (95% CI) | P value |
| --- | --- | --- | --- | --- | --- | --- | --- | --- | --- | --- | --- |
| **Radiological findings** | | | | | | | | | | | |
| Radiolucent line | 15 | 7 | 41.2 (18.4 – 67.1) | 81.5 (71.3 – 89.3) | 31.8 (18.4 – 49.1) | 86.8 (81.4 – 90.9) | 74.5 (64.7 – 82.8) | 2.22 (1.07 – 4.61) | 0.72 (0.48 – 1.09) | 3.1 (1.0 – 9.4) | 0.042 |
| Implant breakage | 22 | 5 | 29.4 (10.3 – 56.0) | 72.8 (61.8 – 82.1) | 18.5 (9.11 – 34.0) | 83.1 (77.9 – 87.3) | 65.3 (55.0 – 74.6) | 1.08 (0.48 – 2.45) | 0.97 (0.69 – 1.35) | 1.1 (0.4 – 3.5) | 0.532 |
| Implant displacement | 9 | 5 | 29.4 (10.3 – 56.0) | 88.9 (80.0 – 94.8) | 35.7 (17.5 – 59.2) | 85.7 (81.4 – 89.2) | 78.6 (69.1 – 86.2) | 2.65 (1.01 – 6.91) | 0.79 (0.58 – 1.09) | 3.3 (1.0 – 11.7) | 0.064 |
| Periosteal reaction | 9 | 6 | 35.3 (14.2 – 61.7) | 88.9 (80.0 – 94.8) | 40.0 (21.5 – 61.9) | 86.8 (82.0 – 90.4) | 79.6 (70.3 – 87.1) | 3.18 (1.30 – 7.74) | 0.73 (0.51 – 1.04) | 4.4 (1.3 – 14.7) | 0.022 |
| **Non-radiological findings** | | | | | | | | | | | |
| Increased WBC count^2^ | 1 (56 cases) | 1 (13 cases) | 7.7 (0.2 – 36.0) | 98.2 (90.5 – 100.0) | 50.0 (6.3 – 93.7) | 82.1 (79.6 – 84.3) | 81.2 (69.9 – 89.6) | 4.31 (0.29 – 64.44) | 0.94 (0.80 – 1.10) | 4.6 (0.3 – 78.6) | 0.343 |
| Increased CRP level^3^ | 4 (56 cases | 6 (13 cases) | 46.1 (19.2 – 74.9) | 92.9 (82.7 – 98.0) | 60.0 (33.0 – 82.0) | 88.1 (81.7 – 92.5) | 84.1 (73.3 – 91.8) | 6.46 (2.13 – 19.65) | 0.58 (0.35 – 0-96) | 11.1 (2.5 – 49.5) | 0.002 |
| Positive tissue cultures | 12 (76 cases) | 14 (17 cases) | 82.4 (56.6 – 96.2) | 84.2 (74.0 – 91.6) | 53.9 (39.9 – 67.2) | 95.5 (88.4 – 98.4) | 83.9 (74.8 – 90.7) | 5.22 (2.97 – 9.17) | 0.21 (0.07 – 0.59) | 24.9 (6.2 – 100.0) | < 0.0001 |
| Positive sonication culture | 21 | 15 | 88.2 (63.6 – 98.5) | 74.1 (63.1 – 83.2) | 41.7 (32.2 – 51.8) | 96.8 (89.0 – 99.1) | 76.5 (66.9 – 84.5) | 3.4 (2.27 – 5.11) | 0.16 (0.04 – 0.59) | 21.4 (4.5 – 101.7) | < 0.0001 |
| Combined microbiology^4^ | 0 | 15 | 88.2 (63.6 – 98.5) | 100.0 (95.6 – 100.0) | 100.0 | 97.6 (91.7 – 99.3) | 98.0 (92.8 – 99.8) | - | 0.12 (0.03 – 0.43) | 1,010.6 (46.2 – 22,090.7) | < 0.0001 |

NOTE. PPV = Positive predictive value; NPV = Negative predictive value; PLR = Positive likelihood ratio; NLR = Negative likelihood ratio; DOR = Diagnostic odds ratio.

^(1)^ Where not otherwise indicated

^(2)^ White blood cell (WBC) count ≥ 11.000/ mm^3^

^(3)^ C-reactive protein (CRP) value ≥ 10 mg/l

^(4)^ Combination of sonication and tissue cultures
